# Generating Real-World Evidence on the Quality Use, Benefits and Safety of Medicines in Australia: History, Challenges and a Roadmap for the Future

**DOI:** 10.3390/ijerph182413345

**Published:** 2021-12-18

**Authors:** Sallie-Anne Pearson, Nicole Pratt, Juliana de Oliveira Costa, Helga Zoega, Tracey-Lea Laba, Christopher Etherton-Beer, Frank M. Sanfilippo, Alice Morgan, Lisa Kalisch Ellett, Claudia Bruno, Erin Kelty, Maarten IJzerman, David B. Preen, Claire M. Vajdic, David Henry

**Affiliations:** 1 Centre for Big Data Research in Health, Faculty of Medicine and Health, UNSW Sydney, Sydney 2052, Australia; j.costa@unsw.edu.au (J.d.O.C.); h.zoega@unsw.edu.au (H.Z.); c.bruno@unsw.edu.au (C.B.); claire.vajdic@unsw.edu.au (C.M.V.); 2 Quality Use of Medicines and Pharmacy Research Centre, Clinical and Health Sciences, University of South Australia, Adelaide 5000, Australia; nicole.pratt@unisa.edu.au (N.P.); lisa.kalisch@unisa.edu.au (L.K.E.); 3 Centre of Public Health Sciences, Faculty of Medicine, University of Iceland, 102 Reykjavik, Iceland; 4 Centre for Health Economics Research and Evaluation, Faculty of Health, University of Technology Sydney, Sydney 2006, Australia; Tracey.Laba@chere.uts.edu.au; 5 WA Centre for Health and Ageing, Medical School, University of Western Australia, Perth 6009, Australia; Christopher.Etherton-Beer@uwa.edu.au (C.E.-B.); frank.sanfilippo@uwa.edu.au (F.M.S.); erin.kelty@uwa.edu.au (E.K.); david.preen@uwa.edu.au (D.B.P.); 6 Research School of Population Health, College of Health and Medicine, Australian National University, Canberra 2601, Australia; alice.morgan@anu.edu.au; 7 Centre for Cancer Research and Centre for Health Policy, Melbourne School of Population and Global Health, University of Melbourne, Melbourne 3000, Australia; maarten.ijzerman@unimelb.edu.au; 8 Institute for Evidence Based Healthcare, Bond University, Gold Coast 4229, Australia; dhenry@bond.edu.au

**Keywords:** prescribing, quality use of medicines, medication safety, pharmacoepidemiology, medication data, data linkage, health outcomes, real-world data, real-world evidence

## Abstract

Australia spends more than $20 billion annually on medicines, delivering significant health benefits for the population. However, inappropriate prescribing and medicine use also result in harm to individuals and populations, and waste of precious health resources. Medication data linked with other routine collections enable evidence generation in pharmacoepidemiology; the science of quantifying the use, effectiveness and safety of medicines in real-world clinical practice. This review details the history of medicines policy and data access in Australia, the strengths of existing data sources, and the infrastructure and governance enabling and impeding evidence generation in the field. Currently, substantial gaps persist with respect to cohesive, contemporary linked data sources supporting quality use of medicines, effectiveness and safety research; exemplified by Australia’s limited capacity to contribute to the global effort in real-world studies of vaccine and disease-modifying treatments for COVID-19. We propose a roadmap to bolster the discipline, and population health more broadly, underpinned by a distinct capability governing and streamlining access to linked data assets for accredited researchers. Robust real-world evidence generation requires current data roadblocks to be remedied as a matter of urgency to deliver efficient and equitable health care and improve the health and well-being of all Australians.

## 1. Introduction

Prescribing medicines is the most common health intervention globally [1]. Modern medicines have changed the course of major diseases including coronary atherosclerosis, heart failure, stroke, HIV/AIDS and several cancers. However, these major advances have come with costs, both human and financial. Medicines are approved by regulators and payers based on evidence from randomised clinical trials (RCTs) [2,3] but most RCTs are not designed to anticipate, identify, or quantify all possible safety concerns, particularly rare outcomes and long-term effects that only emerge once large numbers of people are exposed over time [4,5]. Moreover, RCTs most commonly focus on single medicines and do not necessarily reflect how medicines are used in patients with complex needs, who require multiple medicines for long periods [4,5]. So, when regulatory and subsidy decisions are made, policy makers, health care professionals, and patients face significant uncertainty about whether the benefits and safety reported in these trials will translate into real-world settings. As such, there is a critical need for rapid and comprehensive evidence about the populations accessing new products, and the benefits and harms associated with their use in routine clinical care.

In addition to generating evidence about the effectiveness and safety of medicines, it is imperative that real-world evidence addresses how medicines are used in routine practice. This is because inappropriate prescribing practices may lead to harm, significant downstream health system burden, and waste of health care resources. In Australia, it is estimated that some 2–3% of all hospital admissions are related to medicine use, rising to 20–30% in people aged 65 years and over [6]. In the period 2016–2017, this equated to ~250,000 hospital admissions, estimated conservatively to cost more than $1.3 billion [6]. Moreover, the high unit cost of some medicines can impact on affordability, resulting in inequities in access and health outcomes.

During the last 20 years, the capacity to generate real-world data on quality use, benefits and safety of prescribed medicines has expanded greatly. The field of pharmacoepidemiology has developed from a primary interest in drug utilisation and ecological exposure-outcome studies to contemporary use of large databases of multiple linked routinely collected, real-world data to estimate the balance between the benefits and harms of medicines [7,8]. These analyses have become increasingly important in decision making. For instance, real-world studies of medicines for COVID-19 have profoundly affected our understanding of the positive and negative impacts of these interventions [9,10]. While insights generated from large databases have the potential to augment our understanding of the impacts of health care interventions, poorly conducted, and even fraudulent studies have important consequences that have led to inappropriate, worthless or harmful treatments being administered to millions of people [11].

Never has there been a more important time to shine the spotlight on Australia’s capacity to conduct high-quality, real-world studies of medicine use and effects across a wide range of therapies. In this review, we discuss the use of routine ‘medication data’ to generate insights and enhance our understanding of the real-world use, benefits and safety of medicines in Australia. In this context, we refer to ‘medication data’ as an all-encompassing term that includes prescription, dispensing, sales and self-report data about medicine use. Specifically, we will:

- Discuss Australian medicines policies and detail the available medication data that can be leveraged to estimate real-world medicine use;
- Describe how medication data have been used for population-level monitoring, evaluation and research on quality use, effectiveness and safety of medicines, including a COVID-19 case study;
- Highlight the key barriers to delivering a comprehensive research program quantifying real-world use, effectiveness and safety of medicines in Australia; and
- Outline a roadmap to bolster Australia’s capacity to accelerate evidence development about effectiveness, safety and quality use of medicines in routine clinical care.

## 2. Australian Medicines Policies

Australia has a long history of innovation in medicines policies and Australians can access medicines in a variety of ways. They can be prescribed in the community or to patients during hospital stays. Other medicines, including complementary and alternative medicines, can be purchased over the counter (OTC), without a prescription, in community pharmacies or retail stores such as supermarkets.

### 2.1. National Formulary (The Pharmaceutical Benefits Scheme)

The Pharmaceutical Benefits Scheme (PBS), established in 1948, is a key pillar of Australia’s universal health care system, providing all Australian citizens and permanent residents with subsidised access to prescribed medicines [12]. The Repatriation Pharmaceutical Benefits Scheme (RPBS), established in 1919 provides subsidised access to pharmaceuticals to veterans and their dependents. All PBS medicines are available on the RPBS, but eligible veterans’ and their dependents have access to additional medicines via the RPBS.

The Pharmaceutical Benefits Advisory Committee (PBAC), formed in the early 1950s, is an independent expert body appointed by the Australian Government recommending new medicines for PBS-listing based on clinical efficacy, safety and cost-effectiveness (‘value for money’) relative to other available treatments, a process underpinned by RCT evidence. The PBAC pioneered ‘value for money’ as a pre-requisite for listing in the early 1990s, a process now adopted by governments and third-party payers worldwide [13]. Prior to PBS-listing, a medicine must first be assessed for its quality, efficacy and safety and registered for use in Australia by the Therapeutic Goods Administration (TGA), part of the Australian Government Department of Health. The TGA is responsible for the regulation, registration and ongoing monitoring of medicines safety. Historically, the TGA has relied on periodic review of passive voluntary reports of adverse events from the pharmaceutical industry, prescribers and patients to generate ‘signals’ for investigation. It is widely acknowledged that this system alone does not meet the needs of a contemporary regulatory system [2,3] and there is a critical need for large-scale and comprehensive post-marketing studies leveraging quality real-world data.

### 2.2. Quality Use of Medicines (QUM)

The late 1990s and early 2000s also saw the development of pivotal initiatives promoting quality use of medicines (QUM) in Australia. The Australian Government launched the National Medicines Policy [14] and Australia remains one of the few developed countries detailing a comprehensive approach to produce *better health outcomes for all Australians*, focusing on people’s access to, and wise use of, medicines. The National Prescribing Service (now *NPS MedicineWise*, Sydney, Australia), a not-for-profit organisation funded by the Commonwealth Department of Health, was launched in 1998. Considered the main implementation arm of the National Medicines Policy, the organisation disseminates evidence-based information and implements educational programs to improve the way in which medicines are prescribed and used in Australia [15]. The *Veterans’ Medicines Advice and Therapeutics Education Services* (Veterans’ MATES, Adelaide, Australia) program commenced in 2004 to improve the use of medicines and health services in the veterans’ community through data-driven health interventions directed to both Department of Veterans’ Affairs clients and their health care providers [16]. Most recently, QUM and medicines safety was made the 10th National Health Priority Area by the Council of Australian Governments (COAG) Health Council [17], recognising the urgent need for a coordinated national approach in identifying and promoting best-practice models and measuring progress towards reducing medication related harm.

### 2.3. A Growing Need for Real-World Data

Given Australia’s significant investment in prescribed medicines and QUM initiatives it is imperative that real-world medicines use is monitored to ensure appropriate, effective and safe use. This requires access to comprehensive multiple linked datasets and the capability to perform sophisticated analyses using these data. Cooperation and clear, ongoing governance arrangements between government agencies and academic and not-for-profit institutions are needed to achieve these ends. In the following sections, we detail the diversity of data sources and capabilities needed in Australia to achieve this goal.

## 3. Quantifying Medicines Use in Australia

Australia is replete with medication data to estimate individual- and population-level medicine use, but the available data remain largely unlinked to information that is needed to gain insights into indications for treatment and health outcomes. Primary data collections (e.g., cross-sectional and longitudinal surveys and disease- or medicine-specific registries) and secondary or routinely collected data (e.g., medicine sales, electronic health records, and dispensing claims) have demonstrated utility in describing medicines use in Australia (Table 1). However, the population coverage and the extent of clinical, dosage, and sociodemographic information varies by data source. This poses challenges for comprehensive quantification of medicine use (including, potential underuse, overuse and misuse) across the entire population and especially in population sub-groups, all of which are critical for QUM assessment. For this reason, we limit our discussion in this paper to potentially linkable data from dispensing and prescription records that provide population-wide metrics.

### 3.1. Data from Dispensing Records

Records generated when PBS or RPBS prescriptions are dispensed in pharmacies are the mainstay of Australia’s routinely collected whole of population medication data. These are electronically generated by systems that have low error rates as they record transactions and attract reimbursements for dispensing pharmacies. PBS records have been shown to accurately reflect prescribed medicine use compared to self-reported use and for medicines prescribed and administered in hospital outpatient settings [18,19]. PBS data have proven an invaluable source of information to quantify population-level medicine use and associated outcomes [7,20]. Notwithstanding the strengths and insights that can be generated from data of this kind, they were not established for research purposes and the gaps in, and limitations of, these data to support QUM research must be acknowledged and addressed. For instance, data on indication and directions for use such as the prescribed daily dose are not available in PBS or RPBS records. In some instances, this can be inferred for medicines used for a single indication, with specific patterns of use, or by linking to other data sources. However, researchers are often required to rely on crude approaches to derive estimates for daily doses, adherence, persistence, and concurrent use when analysing these data.

### 3.2. Data from Health Records

The growth in access to electronic health records (EHRs) has also contributed significantly to the discipline of pharmacoepidemiology [21]. The value of these collections lies in the richness of the longitudinal data they contain across sociodemographic, behavioural (e.g., smoking status, alcohol consumption) and clinical (e.g., diagnostic, laboratory, prescribing and imaging) domains. These systems have high quality records on all prescriptions written (including indications and directions for use), irrespective of whether they are publicly subsidised or paid in full by patients [22]. However, the quality and comprehensiveness of sociodemographic, diagnostic and other clinical information are variable. Australia does not have a population-wide EHR. The Australian Institute of Health and Welfare (AIHW) was funded in 2018 to develop an enduring National Primary Care Data Asset; however, consultation about the establishment of this asset is ongoing [23]. The Australian Digital Health Agency also rolled out the national My Health Record system in 2018. This is a personally controlled digital health information summary that can be accessed by individuals and health care providers and connects clinical and administrative information on medical encounters, hospitalisations, imaging services, prescriptions, and pathology results. While the system undoubtedly has value, particularly the retrieval of medical data from a central repository during an emergency, a framework for secondary use of those data (e.g., for research) has not been implemented [24,25].

There is no shortage of data available in Australia to quantify and track medicines use. However, there is no comprehensive data source capturing the full spectrum of medicines purchased and consumed by Australians. Moreover, quantifying and capturing changes in medicine use as people transition to different health care settings (for example, from hospital into community or residential aged care) are challenging, due to the siloed nature of the available data collections.

### 3.3. Difficulties Accessing Linked Person-Level Data in Australia

Maximising the value of Australia’s health data for comprehensive understanding of QUM and real-world effectiveness and safety of medicines has also had many challenges. At the heart of this issue is timely data access. Complexities arising from cross-jurisdictional data linkage across the Commonwealth, States and Territories and concerns about personal privacy are at the heart of the problem and have impacted significantly on the accessibility and timeliness of these data to generate pharmacoepidemiological research to inform medicines policy development in Australia. The federated health system, where the Commonwealth or States and Territories are responsible for specific aspects of care, means some health data collections are under the custodianship of different agencies across different jurisdictions with different legislation. To undertake comprehensive research on medicine effects, medicines exposure data held by the Commonwealth must be linked with outcomes of interest such as hospitalisation or mortality data that are under State and Territory custodianship. While Australia has invested heavily in its data linkage capability, with data linkage units in all jurisdictions [26] and a cross-jurisdictional capability, we have recently highlighted the complex multi-jurisdictional governance processes that limit comprehensive access to linked health data in Australia [27].

## 4. Applications of Medication Data in Australia

### 4.1. Tracking Prescription Medicines Expenditure and Use

The most comprehensive figures on medicine expenditures in Australia, generated by the AIHW, show an annual spend of over $22 billion on prescribed and OTC medicines in the period 2017–2018 for a population of 25 million individuals (this figure includes spending by government, the non-government sector and individuals) [28]. PBS medicines accounted for $11.9 billion of total expenditure; medicines prescribed to public hospital in-patients, private prescriptions and OTC purchases accounted for the remainder. Approximately half of the $3.7 billion spent on medicines purchased OTC was for complementary and alternative medicines. These high-level aggregate figures, however, do not provide insights about individual-level medicine use or QUM. Our recent analysis using individual-level PBS claims in 2018 estimated more than 35% Australians are taking at least one prescribed medicine daily and almost 10% are taking five or more daily [29]. These estimates under-ascertain overall medicine use in our population as they do not include private prescriptions, in-hospital, OTC and complementary and alternative medicines; however, they do generate insights from Australia’s largest publicly funded scheme. These analyses and the analytic code underpinning these estimates could be applied to the most contemporary PBS data to generate publicly available up-to-date snapshots of Australian medicine for the information of governments, researchers and the general public.

### 4.2. Population-Level Monitoring and Evaluation

Many Australian government agencies use medication data routinely to monitor population-level medicine use and outcomes (Table 2). The Drug-Utilisation Sub-Committee (DUSC) of the PBAC was established in 1989 to monitor medicines use post-subsidy (particularly in the first 2 years after PBS listing) and to address specific issues related to QUM. For a period of approximately 20 years DUSC published an annual report, *The Australian Statistics on Medicines (ASM)*, estimating total community use of prescribed medicines (i.e., prescribing outside public hospitals) and detailing prescriptions dispensed according to individual PBS items. Underpinning the publication was a database comprising prescriptions submitted to Medicare Australia for payment of a PBS or RPBS subsidy and estimates of non-subsidised prescriptions; the latter being PBS prescriptions that are under the PBS general beneficiary co-payment and private prescriptions. Estimates of the non-subsidised market were derived from a regular survey of community pharmacies conducted by the Pharmacy Guild of Australia. However, this survey ceased during 2012, coinciding with the collection of unit-record data on under co-payment PBS prescriptions. The last ASM was published in 2016. Since 2003, the Australian Department of Health has generated reports on the number of PBS prescriptions dispensed annually and the total cost to government. However, this is not at the same level of granularity as the ASM, with only the most frequently dispensed PBS medicines and medication classes monitored over consecutive years [30].

The DVA (through their Veterans’ MATES program) and NPS MedicineWise use medication data to target feedback to prescribers to improve QUM. Both programs have demonstrated that these interventions have led to improved medicines use and health outcomes [16,31]. The AIHW also uses a wide range of health data, including PBS, to generate authoritative information and statistics on health and welfare topics. It publishes contemporary snapshots of medicine use, like those cited in the previous section of this review.

The Australian Commission on Safety and Quality in Health Care (ACSQHC) uses PBS and RPBS data to generate the Australian Atlas of Healthcare Variation, monitoring and making recommendations to curtail unwarranted variations in medicine use [32,33,34]. The ACSQHC also hosts the Antimicrobial Use and Resistance in Australia (AURA) Surveillance System, using medication data from various sources to monitor the rate and appropriateness of antimicrobial use in Australia.

The Australian Government National Real-Time Prescription Monitoring (RTPM) system is administered by health departments in each State and Territory. It provides prescribers and pharmacists with up-to-date histories of patients’ supply and prescription of controlled substances, including pain medicines such as oxycodone, morphine and fentanyl and other high-risk medicines (determined within each Australian State or Territory), including all benzodiazepines such as diazepam [35]. In addition, the national Prescription Shopping Program provides doctors with data about patients who are at risk of harm because they have multiple medicines prescribed by different doctors.

### 4.3. Limitations of Current Use of Medication Data in Monitoring and Evaluation

While there is an abundance of activity leveraging medication data across government to monitor the success of Australia’s policies, it is striking that they have focused almost solely on estimating and reporting medicine use based on volume and cost. Consequently, assessment or routine reporting about whether this significant investment delivers better health outcomes for our population, as stipulated in our National Medicines Policy, is lacking [14]. Key exceptions are the programs delivered by Veterans’ MATES and NPS MedicineWise. In the following sections, we explore the challenges in delivering comprehensive evaluation of the impact of medicine.

## 5. Medication Data for Research

Internationally, the scientific discipline of pharmacoepidemiology has burgeoned over the last 20 years, driven by a growing interest in the generation of evidence of real-world effects of medicines and assisted by improved access to individual-level linked health data and methods supporting robust causal inferences from those data [5,36,37,38,39]. Initially, studies focused on serious adverse effects of specific medicine classes, for instance anti-inflammatory agents and antimicrobials. However, continuing improvements in analytic techniques to reduce selection biases and confounding have enabled studies that estimate treatment benefits equivalent to those seen in large RCTs [40,41].

### Characteristics of Australian Pharmacoepidemiological Research Studies

Our systematic reviews [7,20] cataloguing peer-reviewed publications using PBS claims in the period 1987–2018 demonstrate that the vast majority of Australian pharmacoepidemiology research has used aggregate, unliked individual-level PBS, or RPBS data for utilisation studies or to investigate prescriber practice (guideline concordant) or patient behaviour (adherence to treatment) [42,43,44,45,46,47,48]. These studies typically investigated medicines acting on the nervous system (opioids, psychotropics) or for treating cardiovascular disease (statins, antihypertensives, and antithrombotics). Many of these studies have been undertaken in DVA clients or people receiving government benefits exclusively (e.g., PBS concessional beneficiaries). However, studies using the entire PBS-eligible population have increased with the availability of under co-payment data in the PBS collections since 2012. Moreover, the number of studies using individual-level PBS data has accelerated in the last decade due to the availability, to the research community, of a standardised, de-identified data collection of person-level dispensing claims for a 10% sample of PBS eligible people (“PBS 10%”) [7].

Table 3 details published research using PBS claims to assess medicine-related outcomes. We included the studies identified in our previous systematic reviews and also updated the literature searches, using the same methods, to identify medicine-use outcome studies published in 2019 and 2020. Our synthesis of the 107 studies published from 1987 to 2020 identified two main methodological approaches to assess health outcomes associated with medicine use (see the Supplementary File S1 for the list of included studies). First, ecological studies using aggregated data, whereby trends in medicines use were correlated with trends in outcome rates within the same population. This meant that they assessed population-level outcomes rather than examining individual effects of medicines. The ecological studies investigated clinical outcomes, such as mortality, overdose and poisoning, and were most often generated from publicly available data. Second, studies based on person-level and linked data, which addressed medicines safety outcomes such as infections, development of other health conditions, birth defects, hospitalisations (e.g., for myocardial infarction, pneumonia, falls, and fractures, and death) [49,50,51,52,53]. Studies examining effectiveness measured mostly survival or hospitalisations for specific conditions (e.g., heart failure rehospitalisation following post-discharge beta-blocker initiation) [54].

Over half of the studies leveraging individual-level data were undertaken in the DVA population. As a single payer for all health services provided to their clients, the necessary individual-level medicine exposure and outcomes data are readily available without the need for the complex and time-consuming linkage of data across jurisdictions. While these studies have generated important insights about medicine-related safety they are mostly limited to older Australians and focused primarily on medicines used commonly in older populations such as those acting on the nervous (43%) and cardiovascular (22%) systems. Population-based studies exploring medicine use and outcomes according to Aboriginal and Torres Strait Islander status or for people with a disability, from culturally and linguistically diverse (CaLD) backgrounds and refugees are notably absent. Importantly, Australian data have been used in six [38,55,56,57,58,59] global studies investigating medicine-related health outcomes, using novel statistical techniques that evaluate the sequence of medicines dispensed to identify medicine-adverse events. Another 26 have focused on utilisation patterns to benchmark medicine use in Australia against other countries, such as medicines for attention-deficit hyperactivity disorder (ADHD) [44], antipsychotics [60], and antiepileptics [61].

By international standards the number of individual-level medicine use and outcomes studies conducted in Australia is small [37] and certainly not delivering on its potential given the wealth of data available in this country. Nor does this align with the central tenet of Australia’s National Medicines Policy, ensuring we are delivering better health outcomes for our population. We lag far behind other jurisdictions who have joined forces to deliver large-scale global studies of medicine effects to support the evidentiary needs of regulators and payers [62,63,64]. The case study in Box 1 clearly demonstrates how our current infrastructure and data access operating models are ill-equipped to respond rapidly to emerging questions around the real-world impact of repurposed or newly developed treatments to prevent and manage COVID-19.

Box 1 **Australian medication data in the spotlight: Lessons from the COVID-19 pandemic.**

The escalating SARS-CoV-2 case numbers worldwide have highlighted the urgent need for timely, robust evidence about the impact of repurposed or newly developed treatments to prevent and manage COVID-19. Evidence from RCTs evaluating the efficacy and safety of vaccines and disease-modifying agents is accumulating [65,66]. However, the speed of emerging viral variants and the related clinical and policy questions about therapies far outpace the capability to conduct new trials and deliver timely answers to these pressing questions. Moreover, each jurisdiction is unique in terms of disease incidence, vaccination availability and uptake, medicine access, prescriber preferences and policy responses. As such, even when trial evidence is published, it is imperative to track the use of these therapies and quantify their effectiveness and harms as they are rolled out across health systems globally. To achieve this, jurisdictions need robust and agile data infrastructure linking individual-level prescription (or dispensing) data to COVID-19 notifications, hospital data, vaccine and death registries plus accurate, meaningful sociodemographic information to inform efficient and equitable public health responses.

Despite the growth in high-quality, real-world evidence addressing emergent clinical questions about vaccines and medicines across the globe [67,68,69], Australia has been silent on these issues. While some Australian population-based studies are emerging around the changes in prescribed medicine use during the pandemic [70,71,72,73,74,75], none address questions of significant public interest regarding the effectiveness and safety of therapies for COVID-19. This issue has become even more pressing with the emergence of the SARS-CoV-2 Delta and Omicron variants. In the UK, for example, researchers and analysts at Public Health England produce regularly updated high-quality studies answering these critical questions at a national level [76,77]. In Australia, we have all the data elements necessary to conduct these studies, including a newly established national COVID-19 registry [78], but the data required to address these questions remain unlinked and out of reach of health agencies and researchers.

Below, we highlight some further pressing questions regarding the risk factors, clinical progress, prevention, amelioration and treatment of infections by the SARS-CoV-2 variants.

(1) What are the current major determinants of risk of developing severe disease after infection with the Delta variant? How is this changing over time and how do the risk factors compare with the earlier viral strains?
(2) What proportion of patients suffering from COVID-19, and being managed in the community, are receiving adequate evidence-based treatments?
(3) How many individuals receiving unproven, in effective or harmful COVID-19 treatments? This includes, but is not limited to, ivermectin, azithromycin, vitamin D, zinc and quinine derivatives.
(4) What are the socioeconomic factors that determine access to vaccines and how can these population sub-groups most rapidly and effectively be targeted?
(5) How well are the current vaccines (Pfizer, AstraZeneca, and Moderna) working against the Delta virus strain in Australia (in preventing infection, transmission, hospitalisation, ICU admission and death)?
(6) What is the comparative safety of the AstraZeneca and mRNA vaccines (Pfizer and Moderna) in terms of acute sensitivity reactions, thrombocytopenia/venous thrombosis, heart attacks, strokes and myocarditis? In Australia, how do these vaccine-associated risks compare with the risks of acquiring COVID-19?
(7) How should the limited supply of new and expensive monoclonal antibody treatments, now available for treatment of mild to moderate COVID-19 outside hospital, be targeted to those most likely to benefit? Should they be combined with other therapies, e.g., inhaled or oral corticosteroids?
(8) Will the early use of monoclonal antibodies in Australia reduce pressure on the hospital systems?

As a matter of urgency, we propose the creation of a resilient data infrastructure [79] needed to address the questions outlined above. This will enable researchers and governments to respond rapidly to emerging information needs around the evolving pandemic and other major public health challenges.

The pandemic has heightened the aspirations of the international pharmacoepidemiology community to provide much needed evidence in this global public health crisis. However, the publication of poor-quality studies, some of which are based on fraudulent or flawed data, has also exacerbated criticisms that studies of this kind are not reliable or trustworthy [10]. Robust data infrastructure is a key building block to deliver evidence complementing RCTs, however, this must be accompanied by international best-practice principles of transparent and reproducible reporting.

## 6. Key Barriers to Delivering a Comprehensive Program on Real-World Use, Effectiveness and Safety of Medicines in Australia

Our analysis of the peer-reviewed literature examining the outcomes associated with PBS medicine use clearly demonstrates the mismatch between Australia’s annual multi-billion-dollar investment in prescribed medicines and capability to deliver a comprehensive program evaluating the health benefits and harms derived from this investment. There have been a series of high-profile reviews, including the Productivity Commission Report on Data Availability and Use [80] and the Senate Select Committee on Health Sixth Interim Report, Big Health Data: Australia’s Big Potential [81] documenting the contemporary challenges facing the research, government and business sectors in realising the potential of Australian data and recommending responses to turn this situation around (Box 2).

Box 2 **Historical challenges to data availability and use in Australia and key recommendations (dot points) from the Productivity Commission and Senate Select Committee on Health.**

Privacy and data access concerns and lack of trust in existing data access processes and protections

- Develop risk-based data access framework based on risks associated with different types of data, uses of data and use environments
- Ensure linkage policies and regulations are developed to world’s best-practice standard

Legal, institutional and technical barriers

- Simplify existing legislative framework for data access, standardise data sharing agreements, including those pertinent to States and Territories
- Accredit State and Territory, in addition to Commonwealth, data linkage units to link Commonwealth data with State data collections, subject to comprehensive privacy and security protocols
- Use an open data policy for low-risk de-identified data collections

Lengthy, complex and inefficient approval processes and a culture of risk aversion

- Establish new statutory office holder, with responsibility for enabling effective use of data, oversight, guidance and updating operations
- Designate national interest datasets to enable wider use across and between sectors (public, private, not-for-profit and academia) and jurisdictions
- Increase transparency around government data holdings including clear statements regarding dataset approval processes
- By default, deidentified datasets should be released on an enduring basis

Duplicative efforts of ethics committees

- Reform ethics processes including registration requirements and mutual recognition of approvals from accredited jurisdictions

Costs

- Develop enduring linked data assets for use by multiple end-users including government, researchers and other third parties

In the five years since the publication of these recommendations, the Office of the National Data Commissioner has been established and enabled legislation in the form of the Data Availability and Transparency (DAT) Bill 2020 currently before parliament. The purpose of this Bill is to:

- Implement a scheme authorising and regulating access to Australian Government data (this does not include data collected by State and Territory Governments or My Health Record);
- Authorise public-sector data custodians to share data with accredited users according to specific authorisations, purposes, principles and agreements;
- Establish and specify the functions and powers of the National Data Commissioner as the regulator of the scheme and the National Data Advisory Council as an advisory body to the commissioner; and
- Establish the regulation and enforcement framework for the scheme.

The Bill will be a key enabler to data access and use. However, the timeline as well as ways in which the legislation will be interpreted and implemented remain uncertain. Key to this endeavour is sharing of data across jurisdictional boundaries. While almost every Australian jurisdiction has data sharing pathways in place, they vary in their levels of maturity. In a forward step in July 2021, an intergovernmental agreement on data sharing between Commonwealth, State and Territory governments was signed committing all governments to share data between jurisdictions as a default position, if it can be done securely, safely, lawfully and ethically [82]. While this agreement should provide impetus to improve access across jurisdictional boundaries, it makes no reference to data sharing and use for research. This should be remedied.

### 6.1. Tentative Steps towards Greater Data Access in Australia

New guidance is emerging based on the Five Safes Framework, an internationally recognised approach assessing strategic, privacy, security, ethical and operational risks associated with data sharing or release [83]. The DAT Bill 2020 refers to Data Sharing Principles modelled on the Five Safes Framework. However, the Framework is principles based, and subject to interpretation at the coal face. This results in significant heterogeneity and inconsistency between policy agencies. For data linkage projects, this creates lengthy delays and considerable burdens on data custodians and end-users applying for access. It is well documented that data governance demands and inadequate resourcing within government to directly support data access remain as major challenges to research in this area [81]. Data safe havens that securely house potentially sensitive data are a fundamental pillar of the Framework. The ABS DataLab, the Department of Health’s Enterprise Data Warehouse (EDW), the AIHW Secure Remote Access Environment (SRAE), E-Research Institutional Cloud Architecture (ERICA), and the Sax Institute’s Secure Unified Research Environment (SURE) are examples of these facilities. However, resilient remote-access facilities with fit-for-purpose infrastructure and administrative policies and procedures are yet to be delivered at scale.

### 6.2. Inefficiencies in Ethics Approvals for Research Using Linked Data

The Australian National Health and Medical Research Council (NHMRC) Statement on the Ethical Conduct in Human Research [84] has recently been refreshed and provides explicit, implementable guidance on database and data linkage research. However, there is not yet a national approach to single HREC review for data linkage research. While Australian State and Territory health departments have signed a Memorandum of Understanding for mutual acceptance of ethical and scientific review of multi-centre human research projects undertaken in public health organisations, projects involving access to state-wide data collections from every jurisdiction are not included, meaning researchers must navigate duplicative and often inconsistent requirements to gain approval for data linkage studies. Therefore, health data linkage research continues to lag behind other forms of health research including clinical trials of new therapies, resulting in inefficiencies, duplicated effort, inconsistencies and research waste. The challenges with HREC inefficiencies notwithstanding, the major impediment to timely data access, linkage and use are deficient data governance processes.

### 6.3. Tentative Steps to Create National Linked Data Assets

There have been encouraging moves to develop multi-source enduring linked data assets (MELDAs) of national significance. One key example, that could deliver important insights relating to real-world quality use of medicines, safety and effectiveness research, is the National Integrated Health Services Information (NIHSI) asset [85], developed by and under the custodianship of the AIHW. However, several years on from its establishment, formal policies around third-party access, including to academic researchers, are yet to be established. While this asset has been leveraged within government and NIHISI’s precursor (the National Data Linkage Demonstration Project) was accessed by researchers, they were acting as contractors to government. Despite its great promise, the outputs from this resource have been limited to a few publicly available publications and government reports [86,87,88]. The end result is a situation where (i) considerable government investment has been made to create data resource that is not maximally used, and (ii) a highly trained and skilled workforce is unable to access these valuable and comprehensive enduring linked data for the public good.

## 7. Recommendations to Bolster Australia’s Capacity to Accelerate Evidence Development about Quality Use, Effectiveness and Safety of Medicines in Routine Clinical Care

Box 3 outlines our key recommendations to accelerate pharmacoepidemiological research in Australia and leverage the large and growing volumes of routinely collected data to generate evidence for regulators, payers, clinicians, and patients about how medicines are used and how they perform outside the narrow confines of RCTs.

Box 3 **Recommendations to bolster pharmacoepidemiological research in Australia.**

Scale up and streamline data access and use

- Generate publicly available, contemporary snapshots of Australian medicines use
- Increase availability and streamline access to population-wide PBS unit-record data
- Establish dedicated enduring cross-jurisdictional linked data with access for non-government researchers

Enhance medication data collections

- Include private prescriptions in national dispensing data collections
- Link population-wide dispensing and other administrative data to electronic health records

Our review has highlighted the need to deliver publicly available, contemporary Australian statistics on medicines in a user-friendly, interactive form. This will create new levels of transparency for all QUM stakeholders and significantly reduce the burden of bespoke data requests to the custodians of medication data. Starting with PBS and RPBS data, we need to move beyond simple volume and cost metrics and report person-centric information such as number of people dispensed a specific medicine over a defined period (this could be to the level of PBS item codes). Other jurisdictions, such as Denmark, have paved the way for medicine statistics [89], publicly reporting information on prescribed and OTC medicines dispensed/sold, which can be stratified by year, sex, age groups, geographical area (region) and sector (primary or secondary health care sector).

We also demonstrated, in our catalogue of peer-reviewed research using PBS claims, that the availability of a standardised collection of longitudinal person-level PBS data has contributed to the rapid increase in the number of studies investigating QUM, particularly for those used widely in the community. Available via a contract with Services Australia, the collection dates back to 2005 and is now updated monthly and includes the dispensing history for a 10% sample of PBS-eligible Australians. We strongly advocate for this collection to be scaled up to support robust analyses for all PBS medicines; many of the high-cost medicines available on the PBS are for distinct patient sub-groups (e.g., targeted cancer therapies). The current collection is not fit-for-purpose to examine QUM in these high-cost but relatively small volume therapeutic areas.

To bolster high-quality pharmacoepidemiology research, Australian researchers require access to enduring collections of cross-jurisdictional linked data. We support the establishment of an enduring, regularly updated collection linking, at a minimum, PBS, Medicare Benefits Scheme, hospitalisation, emergency department, cancer, and death records; this collection is essentially the NIHSI with the addition of cancer registry data. While this will not negate the need for purpose-built collections, it will serve a substantive proportion of the pharmacoepidemiological research needs. Over time, such a linkage could be augmented with other medication data and routine data collections. For example, access to individual-level dispensing records for private as well as publicly subsidised prescriptions will support more comprehensive QUM reporting, particularly co-prescribing and multi-medicine use. Moreover, linkage to prescribing data held in primary care electronic health records will provide information about all medicines prescribed (not just those that are publicly subsidised), the indication for prescribing, prescribed daily dose and intended duration of therapy. Coupling dispensing and prescribing data expands opportunities to explore critical issues such as primary non-adherence—when prescriptions are written but never filled [90]. Data enhancements enabling researchers to more accurately identify important population sub-groups will enable sophisticated analyses of social and economic determinants of health [91]. All of these enhancements will enable timely and cost-effective responses to new threats to public health and safety; the situation highlighted in our COVID-19 case study needs to be remedied as a matter of urgency.

## 8. Liberating Australia’s Linked Health Data Assets

In this review, we concentrated primarily on enhancements pertaining directly to QUM and pharmacoepidemiological research. We recognise that pharmacoepidemiology sits within a broader discipline of population health research. Box 4 details a series of recommendations that will bolster Australian population health research more broadly and also benefit the discipline of pharmacoepidemiology. In this context, Australia could learn from mature population health research operating models overseas.

Box 4 **Recommendations to bolster population health research (including pharmacoepidemiology) in Australia.**

Establish a distinct capability governing and streamlining access to linked data assets for accredited researchers

- Convene single independent scientific and ethical review of projects leveraging key data collections
- Centralise governance review on behalf of original data custodians

Promote transparency and reproducibility

- Ensure research protocols, analytical code, and data outputs are disseminated freely and openly

Standardise data and analytic tools

- Use common data models, vocabularies and coding

Build and maintain public trust

- Demonstrate the value of data, including enduring linked assets, to improve health system efficiency and equity and the health and well-being of ALL Australians

### 8.1. International Models for Centralisation and Separation of Data Access for Policy and Research

Mature population health research capabilities in jurisdictions such as the United Kingdom, the European Union and Canada, have evolved differently, but they have some common elements Australia would be wise to adopt to scale up current capabilities [92,93]. They all promote the exchange and access to different types of health data for research, have transparent data governance, data sharing agreements for the specific purpose of research and foster continuous improvements around data quality and interoperability. Critically, they have all created distinct entities managing linked data access for approved researchers, essentially separating data provision for routine reporting functions and informing health policy from research. We believe that centralisation of linked health data in Australian government agencies is appropriate, important and should continue for statistical monitoring, and reporting activities. While agencies such as the Australian Bureau of Statistics (ABS) and AIHW have advanced capabilities and capacity to deliver on many of these functions, they are currently not sufficiently equipped or funded to provide the resources and expertise to meet the contemporary research and evaluation needs of a contemporary federated health system. Moreover, these agencies do not have the capacity and resources to manage the substantial number of data requests for the research community. This is likely to become more acute once the enabling legislation is passed and other long-standing roadblocks detailed in Box 2 have been resolved.

### 8.2. A Roadmap for Australia

A more contemporary operating model for Australian population health research would establish a distinct capability with the primary purpose of data access and use for accredited researchers. Aligning with other approaches internationally, this could be delivered by an independent entity or entities. The capability would function strictly according to the Five Safes principles, satisfy legislative requirements at both Commonwealth, State and Territory levels and at arm’s length to vested interests, political, commercial or other. The capability would require up-front government investment but be implemented with a user-pays pricing model. This proposed operating model aligns with the current Australian data reforms and will enhance research and innovation in population health and reduce the significant amounts of research dollars currently invested in the highly convoluted and slow data linkage landscape.

Under this operating model, existing Accredited Data Authorities would continue their work integrating data across jurisdictional boundaries, but they would provide the new capabilities with the core data infrastructure and receive regular data feeds to update the data for the population they are serving. The data provider would also have a key accountability for transparent and efficient response times, a fundamental requirement for publicly funded research. Moreover, a common data model would be integral to the approach, transforming data into common formats using standardised terminologies and vocabularies. Common data models are rapidly accelerating large-scale population health research across the globe as they facilitate systematic data interrogation using libraries of standard analytic routines [64,94]. Critically, the independent capability would assume responsibility as the data custodian of their holdings, absolving the original data custodians of responsibilities for the downstream use of the data. They would undertake single, independent scientific and ethical review of projects leveraging their data holdings; this would obviate the need for ethical and scientific review of projects by multiple jurisdictional entities.

Data sharing agreements with researchers would specify the range of proposed uses for the data, that data should never be reidentified and that data can never be downloaded from its secure host site or in a format that allows identification of individuals. All analyses and products of the analyses would be risk-assessed before release to researchers for use in publications and other scientific outputs. As a condition of data release, the capability would require research projects to align with the international best-practice principles of transparent reporting to ensure all sectors, including the Australian public, have readily accessible information about the approved uses and products of data access. This could take the form of an open, publicly available register using standardised protocol templates [95,96], similar to the long-standing practice of RCT registration. This level of transparency also advances the goal of reproducibility and facilitates peer-review.

Finally, the capabilities will also have a responsibility for, and play a pivotal role in, maintaining public trust, communicating and educating stakeholders about the benefits (and potential risks) of using data for the public good. Fundamental to this effort is embedding and implementing equity principles to identify, monitor and reduce socioeconomic, cultural, gender and age inequities in medicine and health service use and outcomes [91].

## 9. Conclusions

Australia spends in excess of $20 billion annually on medicines. There is no doubt that this has resulted in significant health gains for individuals and populations, but it has also been accompanied by substantial harm and health care costs. Consequently, QUM and medicine safety were announced as Australia’s 10th National Health Priority in 2019. Throughout this review, we highlighted the significant mismatch between Australia’s annual multi-billion-dollar investment in medicines and our capability to deliver a comprehensive research program evaluating the health benefits and harms derived from this investment. We repeatedly highlighted the deficiencies in data access in Australia and how it lags behind most countries with mature publicly funded health care systems. We pointed to the need to establish centralised or distributed data assets operating under the Five Safes principles which would also support contemporary, collaborative, ethical and reproducible research and government activity in population health. In the context of the COVID-19 pandemic, Australia has been notably absent in the global effort to better understand the real-world impact of repurposed or newly developed treatments to prevent and manage COVID-19. The establishment of widely accessible national health data assets is now a matter of urgency. We urge decision makers to respond to this challenge.

## Figures and Tables

**Table 1:** Data sources estimating individual and population-level medicine use in Australia.

Data Source	Individual-Level	Medicines Captured	Other Data	Examples
Self-report	Yes	Survey specific: prescribed, OTC, complementary, and alternative	Indication for use; medical history, smoking status, BMI, location of residence	Study specific, e.g., National Health Survey, Australian Longitudinal Study on Women’s Health (ALSWH), 45 and Up Cohort Study, Bettering the Evaluation of Healthcare (BEACH)
Registries	Yes	Registry for specific medicines or clinical conditions	Indication for use; medical history, pathology, imaging, smoking status, BMI, location of residence	Disease specific, e.g., Australian National Diabetes Audit Longitudinal Register (ANDA-L), Myeloma and Related Diseases Registry (MRDR), Australian Rheumatology Association Database (ARAD), Australian Register of Clinical Registries
Sales	No, aggregate only	Volume of medicine sold to pharmacies, hospitals, supermarkets	Location of sales	Community pharmacy prescriptions, OTC, complementary and alternative medicine sales data, manufacturer sales, hospital sales
PBS and RPBS dispensing	Yes	R/PBS-listed medicines	Indication for some authority-required medicines, age, sex, beneficiary status, locations of prescriber, pharmacy and beneficiary	PBS and RPBS dispensed medicines from hospital and community pharmacies
Electronic health records	Yes	Medicines administered to hospital in-patients or medicines prescribed in primary care	Indication for use, medical history, pathology, imaging, smoking status, BMI	Hospital: Electronic hospital medication management systems, Hospital discharge summaries Community: General practice clinical software, e.g., Medicine Insight, Melbourne East Monash General Practice Database (MAGNET), GP Population Level Analysis and Reporting (POLAR) Both: My Health Record
Drug surveillance	Yes	Controlled substances	Indication available sometimes	Monitoring of Drugs of Dependence System (MODDS), NSW Controlled Drugs Data Collection (CoDDaC), Real-Time Prescription Monitoring (RTPM)

BMI, body mass index; OTC, over the counter; PBS, Pharmaceutical Benefit Scheme; RPBS, Repatriation Pharmaceutical Benefits Scheme.

**Table 2:** Government monitoring and evaluation activities.

Activity and Examples (in Italics)	Purpose	Medication Data Used
Medicines use (volume, cost) *Drug-Utilisation Sub-Committee (DUSC) of the PBAC; PBS expenditure and prescriptions reports; AIHW*	Tracks changes in volume of medicines dispensed and total expenditure	PBS and RPBS claims, surveys
QUM interventions and evaluation *NPS MedicineWise; Veterans’ MATES*	Improvements in quality of prescribing, improved health outcomes	PBS and RPBS claims, MedicineInsight data
Variations in medicine use *Atlas of Healthcare Variation*	Examine unwarranted variations in use by geographic location	PBS and RPBS claims
Appropriateness of medicine use *Antimicrobial Use and Resistance in Australia (AURA) Surveillance System;* *Real-Time Prescription Monitoring (RTPM); Prescription Shopping Program*	Reduce inappropriate prescribing, use and associated harms	PBS and RPBS claims, National Antimicrobial Prescribing Survey, National Antimicrobial Utilisation Surveillance Program, MedicineInsight data

PBAC, Pharmaceutical Benefits Scheme Advisory Committee; PBS, Pharmaceutical Benefits Scheme; RPBS, Repatriation Pharmaceutical Benefits Scheme; QUM, Quality Use of Medicines.

**Table 3:** Characteristics of Australian studies assessing medicine use and health outcomes (1987–2020).

Characteristic	Studies Using Aggregate Data (*N* = 28) *n* (%)	Studies Using Individual-Level Data (*N* = 79) *n* (%)
Outcome of interest ^§^		
Safety (at least one outcome)	26 (92.9)	65 (82.3)
Mortality	12 (42.9)	8 (10.1)
Hospitalisations	5 (17.9)	37 (46.8)
Overdose or poisoning	11 (39.3)	0 (0.0)
Maternal or birth complications	0 (0.0)	8 (10.1)
Other health events	9 (32.1)	21 (26.6)
Effectiveness (at least one outcome)	2 (7.1)	14 (17.7)
Survival	0 (0.0)	9 (11.4)
Hospitalisations	0 (0.0)	4 (5.1)
Health events	2 (7.1)	2 (2.5)
Data sources		
Dispensing claims only	0 (0.0)	12 (15.2)
Dispensing claims and other health data	28 (100.0)	0 (0.0)
Dispensing claims and other linked health data	0 (0.0)	67 (84.8)
Medicines focus according to ATC level ^§^		
Alimentary tract and metabolism	1 (3.6)	16 (20.3)
Blood and blood forming organs	1 (3.6)	4 (5.1)
Cardiovascular system	3 (10.7)	17 (21.5)
Genito-urinary system and sex hormones	3 (10.7)	7 (8.9)
Systemic hormonal preparations	0 (0.0)	3 (3.8)
Anti-infectives for systemic use	0 (0.0)	2 (2.5)
Antineoplastic and immunomodulating agents	2 (7.1)	9 (11.4)
Antineoplastic	0 (0.0)	8 (10.1)
Immunomodulating agents	2 (7.1)	1 (1.3)
Musculoskeletal system	3 (10.7)	11 (13.9)
Nervous system	14 (50.0)	34 (43.0)
Respiratory system	0 (0.0)	7 (8.9)
Other ATC groups	0 (0.0)	8 (10.1)
All ATC groups	1 (3.6)	13 (59.1)
Publication Year		
1987–2000	1 (3.6)	0 (0.0)
2001–2005	0 (0.0)	1 (1.3)
2006–2010	7 (25.0)	13 (16.5)
2011–2015	8 (28.6)	30 (38.0)
2016–2020	12 (42.9)	36 (45.6)
Study Population: Age profile		
No age restrictions	24 (85.7)	18 (22.8)
Older adults (≥65 years)	0 (0.0)	46 (58.2)
Adults (≥18 years)	3 (10.7)	4 (5.1)
Women of child-bearing age	0 (0.0)	10 (12.7)
Children *	1 (3.6)	1 (1.3)
Study population: Beneficiary status		
All PBS beneficiaries	24 (85.7)	25 (31.6)
Concessional PBS beneficiaries ^⸸^	4 (14.3)	9 (11.4)
Clients of the Department of Veterans’ Affairs	0 (0.0)	45 (57.0)

^§^ Study could be classified under more than one category. * Studies also included adolescents or young adults. ^⸸^ People receiving government benefits and eligible to pay lower PBS co-payment thresholds. PBS, Pharmaceutical Benefits Scheme.

## Data Availability

Data sharing is not applicable to this article.

## Supplementary Materials

The following are available online at https://www.mdpi.com/1660-4601/18/24/13345/s1, File S1: Studies included in this review assessing medicine exposure and outcomes using Pharmaceutical Benefits Scheme or Repatriation Pharmaceutical Benefits Scheme data.
