# Supplementary material for: Generating Real-World Evidence on the Quality Use, Benefits and Safety of Medicines in Australia: History, Challenges and a Roadmap for the Future
Source: Int J Environ Res Public Health. 2021 Dec 18;18(24):13345. doi: 10.3390/ijerph182413345 (PMC8707536; doi:10.3390/ijerph182413345)
Supplement: Supplementary file 1 [file ijerph-18-13345-s001.zip › ijerph-1398632-supplementary.pdf]

Supplementary file

Studies included in the review assessing medicine exposure and outcomes using Pharmaceutical Benefits Scheme or Repatriation Pharmaceutical Benefits Scheme data

1. Ahmed, B.; Tran, D.T.; Zoega, H.; Kennedy, S.E.; Jorm, L.R.; Havard, A. Maternal and perinatal outcomes associated with the use of renin-angiotensin system (RAS) blockers for chronic hypertension in early pregnancy. *Pregnancy Hypertens.* **2018**, *14*, 156-161.
2. Berecki-Gisolf, J.; Hassani-Mahmooei, B.; Clapperton, A.; McClure, R. Prescription opioid dispensing and prescription opioid poisoning: Population data from Victoria, Australia 2006 to 2013. *Aust. N. Z. J. Public Health* **2017**, *41*, 85-91.
3. Berling, I.; Buckley, N.A.; Isbister, G.K. The antipsychotic story: Changes in prescriptions and overdose without better safety. *Br. J. Clin. Pharmacol.* **2016**, *82*, 249–254
4. Blanch, B.; Pearson, S.A.; Haber, P.S. An overview of the patterns of prescription opioid use, costs and related harms in Australia. *Br. J. Clin. Pharmacol.* **2014**, *78*, 1159-1166.
5. Brett, J.; Wylie, C.E.; Raubenheimer, J.; Isbister, G.K.; Buckley, N.A. The relative lethal toxicity of pharmaceutical and illicit substances: A 16-year study of the Greater Newcastle Hunter Area, Australia. *Br. J. Clin. Pharmacol.* **2019**, *85*, 2098-2107.
6. Buckley, N.A.; Whyte, I.M.; Dawson, A.H.; Isbister, G.K. A prospective cohort study of trends in self-poisoning, Newcastle, Australia, 1987-2012: plus ca change, plus c'est la meme chose. *Med. J. Aust.* **2015**, *202*, 438-442.
7. Buckley, N.A.; Whyte, I.M.; Dawson, A.H.; McManus, P.R.; Ferguson, N.W. Correlations between prescriptions and drugs taken in self-poisoning. Implications for prescribers and drug regulation. *Med. J. Aust.* **1995**, *162*, 194-197.
8. Cairns, R.; Daniels, B.; Wood, D.A.; Brett, J. ADHD medication overdose and misuse: the NSW Poisons Information Centre experience, 2004-2014. *Med. J. Aust.* **2016**, *204*, 154.
9. Cairns, R.; Karanges, E.A.; Wong, A.; Brown, J.A.; Robinson, J.; Pearson, S.A.; Dawson, A.H.; Buckley, N.A. Trends in self-poisoning and psychotropic drug use in people aged 5-19 years: a population-based retrospective cohort study in Australia. *BMJ Open* **2019**, *9*, e026001.
10. Cairns, R.; Schaffer, A.L.; Ryan, N.; Pearson, S.A.; Buckley, N.A. Rising pregabalin use and misuse in Australia: trends in utilization and intentional poisonings. *Addiction* **2019**, *114*, 1026-1034.
11. Canfell, K.; Banks, E.; Clements, M.; Kang, Y.J.; Moa, A.; Armstrong, B.; Beral, V. Sustained lower rates of HRT prescribing and breast cancer incidence in Australia since 2003. *Breast Cancer Res. Treat.* **2009**, *117*, 671-673.
12. Canfell, K.; Banks, E.; Moa, A.M.; Beral, V. Decrease in breast cancer incidence following a rapid fall in use of hormone replacement therapy in Australia. *Med. J. Aust.* **2008**, *188*, 641-644.
13. Castle, D.J.; Chung, E. Cardiometabolic comorbidities and life expectancy in people on medication for schizophrenia in Australia. *Curr. Med. Res. Opin.* **2018**, *34*, 613-618.
14. Caughey, G.E.; Kalisch Ellett, L.M.; Goldstein, S.; Roughead, E.E. Suboptimal medication-related quality of care preceding hospitalisation of older patients. *Med. J. Aust.* **2015**, *203*, 220.e221-227.
15. Caughey, G.E.; Preiss, A.K.; Vitry, A.I.; Gilbert, A.L.; Roughead, E.E. Comorbid diabetes and COPD: impact of corticosteroid use on diabetes complications. *Diabetes Care* **2013**, *36*, 3009-3014.
16. Caughey, G.E.; Roughead, E.E.; Pratt, N.; Killer, G.; Gilbert, A.L. Stroke risk and NSAIDs: An australian population-based study. *Med. J. Aust.* **2011**, *195*, 525-529.
17. Caughey, G.E.; Roughead, E.E.; Pratt, N.; Shakib, S.; Vitry, A.I.; Gilbert, A.L. Increased risk of hip fracture in the elderly associated with prochlorperazine: is a prescribing cascade contributing? *Pharmacoepidemiol. Drug Saf.* **2010**, *19*, 977-982.

18. Caughey, G.E.; Shakib, S.; Barratt, J.D.; Roughead, E.E. Use of Medicines that May Exacerbate Heart Failure in Older Adults: Therapeutic Complexity of Multimorbidity. *Drugs Aging* **2019**, *36*, 471-479.
19. Chan, V.; Tett, S.E. How is leflunomide prescribed and used in Australia? Analysis of prescribing and adverse effect reporting. *Pharmacoepidemiol. Drug Saf.* **2006**, *15*, 485-493.
20. Chitty, K.M.; Butterworth, P.; Batterham, P.J. Antidepressant use and its relationship with current symptoms in a population-based sample of older Australians. *J. Affect. Disord.* **2019**, *258*, 83-88.
21. Colvin, L.; Gill, A.W.; Slack-Smith, L.; Stanley, F.J.; Bower, C. Off-Label Use of Ondansetron in Pregnancy in Western Australia. *Biomed Research International* **2013**, *909860*.
22. Colvin, L.; Slack-Smith, L.; Stanley, F.J.; Bower, C. Pharmacovigilance in pregnancy using population-based linked datasets. *Pharmacoepidemiol. Drug Saf.* **2009**, *18*, 211-225.
23. Colvin, L.; Slack-Smith, L.; Stanley, F.J.; Bower, C. Linking a pharmaceutical claims database with a birth defects registry to investigate birth defect rates of suspected teratogens. *Pharmacoepidemiol. Drug Saf.* **2010**, *19*, 1137-1150.
24. Colvin, L.; Slack-Smith, L.; Stanley, F.J.; Bower, C. Dispensing patterns and pregnancy outcomes for women dispensed selective serotonin reuptake inhibitors in pregnancy.[Erratum appears in Birth Defects Res A Clin Mol Teratol. 2011 Apr;91(4):268]. *Birth Defects Research* **2011**, *91*, 142-152.
25. Colvin, L.; Slack-Smith, L.; Stanley, F.J.; Bower, C. Early morbidity and mortality following in utero exposure to selective serotonin reuptake inhibitors: a population-based study in Western Australia. *CNS Drugs* **2012**, *26*, e1-14.
26. Crossin, R.; Scott, D.; Arunogiri, S.; Smith, K.; Dietze, P.M.; Lubman, D.I. Pregabalin misuse-related ambulance attendances in Victoria, 2012-2017: characteristics of patients and attendances. *Med. J. Aust.* **2019**, *210*, 75-79.
27. Damianovich, D.; Adena, M.; Tebbutt, N.C. Treatment of 5-fluorouracil refractory metastatic colorectal cancer: an Australian population-based analysis. *Br. J. Cancer* **2007**, *96*, 546-550.
28. Daniels, B.; Kiely, B.E.; Houssami, N.; Lord, S.J.; Dobbins, T.; Lu, C.Y.; Ward, R.L.; Pearson, S.A. Survival outcomes for Australian women receiving trastuzumab for HER2-positive metastatic breast cancer following (neo)adjuvant trastuzumab: A national population-based observational study (2006-2014). *Br. J. Cancer* **2018**, *118*, 441-447.
29. Daniels, B.; Kiely, B.E.; Lord, S.J.; Houssami, N.; Lu, C.Y.; Ward, R.L.; Pearson, S.-A. Trastuzumab for metastatic breast cancer: Real world outcomes from an Australian whole-of-population cohort (2001–2016). *The Breast* **2018**, *38*, 7-13.
30. Daniels, B.; Kiely, B.E.; Lord, S.J.; Houssami, N.; Lu, C.Y.; Ward, R.L.; Pearson, S.A. Long-term survival in trastuzumab-treated patients with HER2-positive metastatic breast cancer: real-world outcomes and treatment patterns in a whole-of-population Australian cohort (2001-2016). *Breast Cancer Res. Treat.* **2018**, *171*, 151-159.
31. Daniels, B.; Kiely, B.E.; Tang, M.; Tervonen, H.; Pearson, S.A. Trastuzumab use in older patients with HER2-positive metastatic breast cancer: outcomes and treatment patterns in a whole-of-population Australian cohort (2003-2015). *BMC Cancer* **2019**, *19*, 909.
32. Daniels, B.; Pearson, S.A.; Vajdic, C.M.; Pottegård, A.; Buckley, N.A.; Zoega, H. Risk of squamous cell carcinoma of the lip and cutaneous melanoma in older Australians using hydrochlorothiazide: A population-based case-control study. *Basic Clin. Pharmacol. Toxicol.* **2020**, *127*, 320-328.
33. Fisher, A.; Martin, J.; Srikusalanukul, W.; Davis, M. Bisphosphonate use and hip fracture epidemiology: ecologic proof from the contrary. *Clin. Interv. Aging* **2010**, *5*, 355.
34. Fisher AA; O'Brien ED; MW., D. Trends in hip fracture epidemiology in Australia: possible impact of bisphosphonates and hormone replacement therapy. *Bone* **2009**, *45*, 246-253.
35. Hollingworth, S.A.; McGuire, T.M.; Pache, D.; Eadie, M.J. Dopamine Agonists: Time Pattern of Adverse Effects Reporting in Australia. *Drugs Real World Outcomes* **2015**, *2*, 199-203.

36. Hollingworth, S.A.; Ostino, R.; David, M.C.; Martin, J.H.; Tett, S.E. Ezetimibe: Use, costs, and adverse events in Australia. *Cardiovasc. Ther.* **2017**, *35*, 40-46.
37. Hollis J; Grayson D; Forrester L; Brodaty H; Touyz S; R., C. Antipsychotic medication dispensing and risk of death in veterans and war widows 65 years and older. *Am. J. Geriatr. Psychiatry* **2007**, *15*, 932-941.
38. Hollis, J.; Forrester, L.; Brodaty, H.; Touyz, S.; Cumming, R.; Grayson, D. Risk of death associated with antipsychotic drug dispensing in residential aged care facilities. *Aust. N. Z. J. Psychiatry* **2007**, *41*, 751-758.
39. Hollis, J.; Touyz, S.; Grayson, D.; Forrester, L. Antipsychotic medication dispensing and associated odds ratios of death in elderly veterans and war widows, 2001. *Aust. N. Z. J. Psychiatry* **2006**, *40*, 981-986.
40. Huang, W.; Castelino, R.L.; Peterson, G.M. Adverse event notifications implicating metformin with lactic acidosis in Australia. *J. Diabetes Complications* **2015**, *29*, 1261-1265.
41. Jamolowicz, A.I.; Chen, H.Y.; Panegyres, P.K. Statins and memory loss: An Australian experience. *Australasian Medical Journal* **2015**, *8*, 73-79.
42. Jamshidi, N.; Morley, K.C.; Cairns, R.; Dawson, A.; Haber, P.S. A Review of Baclofen Overdoses in Australia: Calls to a Poisons Information Centre and a Case Series. *Alcohol Alcohol.* **2019**, *54*, 73-78.
43. Kalisch Ellett, L.M.; Pratt, N.L.; Le Blanc, V.T.; Westaway, K.; Roughead, E.E. Increased risk of hospital admission for dehydration or heat-related illness after initiation of medicines: a sequence symmetry analysis. *J. Clin. Pharm. Ther.* **2016**, *41*, 503-507.
44. Kalisch Ellett, L.M.; Pratt, N.L.; Ramsay, E.N.; Barratt, J.D.; Roughead, E.E. Multiple anticholinergic medication use and risk of hospital admission for confusion or dementia. *J. Am. Geriatr. Soc.* **2014**, *62*, 1916-1922.
45. Kalisch Ellett, L.M.; Pratt, N.L.; Ramsay, E.N.; Sluggett, J.K.; Barratt, J.D.; Roughead, E.E. Central Nervous System-Acting Medicines and Risk of Hospital Admission for Confusion, Delirium, or Dementia. *J. Am. Med. Dir. Assoc.* **2016**, *17*, 530-534.
46. Kalisch, L.M.; Caughey, G.E.; Barratt, J.D.; Ramsay, E.N.; Killer, G.; Gilbert, A.L.; Roughead, E.E. Prevalence of preventable medication-related hospitalizations in Australia: an opportunity to reduce harm. *Int. J. Qual. Health Care* **2012**, *24*, 239-249.
47. Kassie, G.M.; Kalisch Ellett, L.M.; Nguyen, T.A.; Roughead, E.E. Use of medicines that may precipitate delirium prior to hospitalisation in older Australians with delirium: An observational study. *Australas. J. Ageing* **2019**, *38*, 124-131.
48. Kelty, E.; Tran, D.; Lavin, T.; Preen, D.B.; Hulse, G.; Havard, A. Prevalence and safety of acamprosate use in pregnant alcohol-dependent women in New South Wales, Australia. *Addiction* **2019**, *114*, 206-215.
49. Kelty, E.; Tran, D.D.; Atkinson, A.; Preen, D.B.; Havard, A. Maternal and Neonatal Health Outcomes Associated with the Use of Gliclazide and Metformin for the Treatment of Diabetes in Pregnancy: A Record Linkage Study. *Diabetes Technol. Ther.* **2020**, *22*, 96-102.
50. Kerr, S.J.; Rowett, D.S.; Sayer, G.P.; Whicker, S.D.; Saltman, D.C.; Mant, A. All-cause mortality of elderly Australian veterans using COX-2 selective or non-selective NSAIDs: a longitudinal study. *Br. J. Clin. Pharmacol.* **2011**, *71*, 936-942.
51. King, C.E.; Pratt, N.L.; Craig, N.; Thai, L.; Wilson, M.; Nandapalan, N.; Kalisch Ellet, L.; Behm, E.C. Detecting Medicine Safety Signals Using Prescription Sequence Symmetry Analysis of a National Prescribing Data Set. *Drug Saf.* **2020**, *43*, 787-795.
52. Ko, H.H.T.; Lareu, R.R.; Dix, B.R.; Hughes, J.D.; Parsons, R.W. A sequence symmetry analysis of the interrelationships between statins, diabetes and skin infections. *Br. J. Clin. Pharmacol.* **2019**, *85*, 2559-2567.
53. Kumar, S.S.; McManus, H.; Radovich, T.; Greenfield, J.R.; Viardot, A.; Williams, K.M.; Cronin, P.; Day, R.O. Interrogation of a longitudinal, national pharmacy claims dataset to explore factors that predict the need for add-on therapy in older and socioeconomically

disadvantaged Australians with type 2 diabetes mellitus patients (T2DM). *Eur. J. Clin. Pharmacol.* **2018**, *74*, 1327-1332.
54. Lai, E.C.C.; Shin, J.Y.; Kubota, K.; Man, K.K.C.; Park, B.J.; Pratt, N.; Roughead, E.E.; Wong, I.C.K.; Kao Yang, Y.H.; Setoguchi, S. Comparative safety of NSAIDs for gastrointestinal events in Asia-Pacific populations: A multi-database, international cohort study. *Pharmacoepidemiol. Drug Saf.* **2018**, *27*, 1223-1230.
55. Leach, M.J.; Pratt, N.L.; Roughead, E.E. Medicine use among older Australians before and after hip fracture. *Journal of Pharmacy Practice and Research* **2013**, *43*, 265-268.
56. Leach, M.J.; Pratt, N.L.; Roughead, E.E. Psychoactive medicine use and the risk of hip fracture in older people: a case-crossover study. *Pharmacoepidemiol. Drug Saf.* **2015**, *24*, 576-582.
57. Leach, M.J.; Pratt, N.L.; Roughead, E.E. The Risk of Hip Fracture Due to Mirtazapine Exposure When Switching Antidepressants or Using Other Antidepressants as Add-On Therapy. *Drugs - Real World Outcomes* **2017**, *4*, 247-255.
58. Leach, M.J.; Pratt, N.L.; Roughead, E.E. Risk of Hip Fracture in Older People Using Selective Serotonin Reuptake Inhibitors and Other Psychoactive Medicines Concurrently: A Matched Case-Control Study in Australia. *Drugs - Real World Outcomes* **2017**, *4*, 87-96.
59. Lee, J.; Pilgrim, J.; Gerostamoulos, D.; Robinson, J.; Wong, A. Increasing rates of quetiapine overdose, misuse, and mortality in Victoria, Australia. *Drug Alcohol Depend.* **2018**, *187*, 95-99.
60. Lee, W.S.; Parsons, S.; Cugley, D.; Rogers, S.; Lim, L.L.; Hall, A. Increased incidence of glaucoma medication usage in middle-aged Australian males taking antiretroviral medication - a population-based study. *J Ophthalmic Inflamm Infect* **2020**, *10*, 30.
61. Lopez, D.; Preen, D.B.; Etherton-Beer, C.; Sanfilippo, F.M. Frailty, and not medicines with anticholinergic or sedative effects, predicts adverse outcomes in octogenarians admitted for myocardial infarction: Population-level study. *Australas. J. Ageing* **2021**, *40*, e155-e162.
62. Man, K.K.C.; Shao, S.C.; Chaiyakunapruk, N.; Dilokthornsakul, P.; Kubota, K.; Li, J.; Ooba, N.; Pratt, N.; Pottegård, A.; Rasmussen, L.; et al. Metabolic events associated with the use of antipsychotics in children, adolescents and young adults: a multinational sequence symmetry study. *Eur. Child Adolesc. Psychiatry* **2020**.
63. Mangoni, A.A.; Woodman, R.J.; Gaganis, P.; Gilbert, A.L.; Knights, K.M. Use of non-steroidal anti-inflammatory drugs and risk of incident myocardial infarction and heart failure, and all-cause mortality in the Australian veteran community. *Br. J. Clin. Pharmacol.* **2010**, *69*, 689-700.
64. Mangoni, A.A.; Woodman, R.J.; Gilbert, A.L.; Knights, K.M. Use of non-steroidal anti-inflammatory drugs and risk of ischemic and hemorrhagic stroke in the Australian veteran community. *Pharmacoepidemiol. Drug Saf.* **2010**, *19*, 490-498; DOI:10.1002/pds.1945.
65. McEwen, J.; Purcell, P.M.; Hill, R.L.; Calcino, L.J.; Riley, C.G. The incidence of pancytopenia in patients taking leflunomide alone or with methotrexate. *Pharmacoepidemiol. Drug Saf.* **2007**, *16*, 65-73.
66. Meuleners, L.B.; Duke, J.; Lee, A.H.; Palamara, P.; Hildebrand, J.; Ng, J.Q. Psychoactive medications and crash involvement requiring hospitalization for older drivers: a population-based study. *J. Am. Geriatr. Soc.* **2011**, *59*, 1575-1580.
67. Moffat, A.K.; Apajee, J.; Pratt, N.L.; Blacker, N.; Le Blanc, V.T.; Roughead, E.E. Use of medicines associated with dry mouth and dental visits in an Australian cohort. *Aust. Dent. J.* **2020**, *65*, 189-195.
68. Ng, H.S.; Koczwara, B.; Roder, D.; Vitry, A. Development of comorbidities in men with prostate cancer treated with androgen deprivation therapy: an Australian population-based cohort study. *Prostate Cancer Prostatic Dis.* **2018**, *21*, 403-410.
69. Ng, H.S.; Koczwara, B.; Roder, D.M.; Niyonsenga, T.; Vitry, A.I. Comorbidities in Australian women with hormone-dependent breast cancer: A population-based analysis. *Med. J. Aust.* **2018**, *208*, 24-28.

70. Nguyen, T.A.; Caughey, G.; Pratt, N.; Shakib, S.; Kemp, A.; Roughead, E. Hospitalization for drug-induced hepatotoxicity: linking Y-codes with pharmaceutical claims data to identify implicated medicines. *J. Clin. Pharm. Ther.* **2015**, *40*, 213-219.
71. Pratt, N.; Andersen, M.; Bergman, U.; Choi, N.-K.; Gerhard, T.; Huang, C.; Kimura, M.; Kimura, T.; Kubota, K.; Lai, E.C.C.; et al. Multi-country rapid adverse drug event assessment: the Asian Pharmacoepidemiology Network (AsPEN) antipsychotic and acute hyperglycaemia study. *Pharmacoepidemiology and Drug Safety* **2013**, *22*, 915-924.
72. Pratt, N.; Chan, E.W.; Choi, N.K.; Kimura, M.; Kimura, T.; Kubota, K.; Lai, E.C.; Man, K.K.; Ooba, N.; Park, B.J.; et al. Prescription sequence symmetry analysis: assessing risk, temporality, and consistency for adverse drug reactions across datasets in five countries. *Pharmacoepidemiol. Drug Saf.* **2015**, *24*, 858-864.
73. Pratt, N.; Roughead, E.E.; Ramsay, E.; Salter, A.; Ryan, P. Risk of hospitalization for hip fracture and pneumonia associated with antipsychotic prescribing in the elderly: a self-controlled case-series analysis in an Australian health care claims database. *Drug Saf.* **2011**, *34*, 567-575.
74. Pratt, N.; Roughead, E.E.; Ryan, P.; Gilbert, A.L. Differential Impact of NSAIDs on Rate of Adverse Events that Require Hospitalization in High-Risk and General Veteran Populations A Retrospective Cohort Study. *Drugs Aging* **2010**, *27*, 63-71.
75. Pratt, N.; Roughead, E.E.; Ryan, P.; Salter, A. Antipsychotics and the risk of death in the elderly: an instrumental variable analysis using two preference based instruments. *Pharmacoepidemiol. Drug Saf.* **2010**, *19*, 699-707.
76. Pratt, N.L.; Ramsay, E.; Kalisch Ellett, L.M.; Duszynski, K.; Shakib, S.; Kerr, M.; Caughey, G.; Roughead, E.E. Comparative effectiveness and safety of low-strength and high-strength direct oral anticoagulants compared with warfarin: a sequential cohort study. *BMJ Open* **2019**, *9*, e026486.
77. Pratt, N.L.; Ramsay, E.N.; Kalisch Ellett, L.M.; Nguyen, T.A.; Barratt, J.D.; Roughead, E.E. Association between use of multiple psychoactive medicines and hospitalization for falls: Retrospective analysis of a large healthcare claim database. *Drug Saf.* **2014**, *37*, 529-535.
78. Pratt, N.L.; Ramsay, E.N.; Kalisch Ellett, L.M.; Nguyen, T.A.; Roughead, E.E. Association between Ophthalmic Timolol and Hospitalisation for Bradycardia. *J Ophthalmol* **2015**, *2015*, 567387.
79. Pratt, N.L.; Ramsay, E.N.; Kemp, A.; Kalisch-Ellett, L.M.; Shakib, S.; Caughey, G.E.; Ryan, P.; Graves, S.; Roughead, E.E. Ranibizumab and Risk of Hospitalisation for Ischaemic Stroke and Myocardial Infarction in Patients with Age-Related Macular Degeneration: A Self-Controlled Case-Series Analysis. *Drug Saf.* **2014**, *37*, 1021-1027.
80. Pratt, N.L.; Roughead, E.E.; Ramsay, E.; Salter, A.; Ryan, P. Risk of hospitalization for stroke associated with antipsychotic use in the elderly: a self-controlled case series. *Drugs Aging* **2010**, *27*, 885-893.
81. Price, S.D.; Holman, C.D.; Sanfilippo, F.M.; Emery, J.D. Are high-care nursing home residents at greater risk of unplanned hospital admission than other elderly patients when exposed to Beers potentially inappropriate medications? *Geriatr Gerontol Int* **2013**.
82. Price, S.D.; Holman, C.D.; Sanfilippo, F.M.; Emery, J.D. Association between potentially inappropriate medications from the Beers criteria and the risk of unplanned hospitalization in elderly patients. *Ann. Pharmacother.* **2014**, *48*, 6-16.
83. Price, S.D.; Holman, C.D.; Sanfilippo, F.M.; Emery, J.D. Does ongoing general practitioner care in elderly patients help reduce the risk of unplanned hospitalization related to Beers potentially inappropriate medications? *Geriatr. Geronto.l Int.* **2015**, *15*, 1031-1039.
84. Price, S.D.; Holman, C.D.A.J.; Sanfilippo, F.M.; Emery, J.D. Use of case–time–control design in pharmacovigilance applications: exploration with high-risk medications and unplanned hospital admissions in the Western Australian elderly. *Pharmacoepidemiol. Drug Saf.* **2013**, *22*, 1159-1170.

85. Qin, X.; Hung, J.; Knuiman, M.; Teng, T.H.K.; Briffa, T.; Sanfilippo, F.M. Evidence-based pharmacotherapies used in the postdischarge phase are associated with improved one-year survival in senior patients hospitalized with heart failure. *Cardiovasc. Ther.* **2018**, *36*, e12464.
86. Qin, X.; Hung, J.; Knuiman, M.W.; Briffa, T.G.; Teng, T.K.; Sanfilippo, F.M. Comparison of medication adherence measures derived from linked administrative data and associations with mortality using restricted cubic splines in heart failure patients. *Pharmacoepidemiol. Drug Saf.* **2020**, *29*, 208-218.
87. Ramsay, E.N.; Pratt, N.L.; Ryan, P.; Roughead, E.E. Proton pump inhibitors and the risk of pneumonia: a comparison of cohort and self-controlled case series designs. *BMC Med. Res. Methodol.* **2013**, *13*, 82.
88. Ramsay, E.N.; Roughead, E.E.; Ewald, B.; Pratt, N.L.; Ryan, P. A self-controlled case series to assess the effectiveness of beta blockers for heart failure in reducing hospitalisations in the elderly. *BMC Med. Res. Methodol.* **2011**, *11*, 106.
89. Rintoul, A.; Dobbin, M.; Nielsen, S.; Degenhardt, L.; Drummer, O. Recent increase in detection of alprazolam in Victorian heroin-related deaths. *Med. J. Aust.* **2013**, *198*, 206-209.
90. Roberts, G.; Chong, C.-R.; Quinn, S.; Cameron-Collins, S.; Forbes, H.; Johnson, J.L.; Kitto, L.; Marotti, S.; Nguyen, H.; Reid, S.; et al. Evaluation of the effect of direct oral anticoagulant availability on hospital presentations for bleeding related to oral anticoagulation in South Australia. *Journal of Pharmacy Practice and Research* **2019**, *49*, 517-524.
91. Robinson, D.A.; Ghaly, B.; Hayen, A.; Lusby, R.J. Statin therapy and carotid endarterectomy: a review of trends in New South Wales, 1990-2004. *ANZ J. Surg.* **2009**, *79*, 456-461.
92. Roughead, E.E.; Chan, E.W.; Choi, N.K.; Griffiths, J.; Jin, X.M.; Lee, J.; Kimura, M.; Kimura, T.; Kubota, K.; Lai, E.C.; et al. Proton pump inhibitors and risk of Clostridium difficile infection: a multi-country study using sequence symmetry analysis. *Expert Opin. Drug Saf.* **2016**, *15*, 1589-1595.
93. Roughead, E.E.; Chan, E.W.; Choi, N.K.; Kimura, M.; Kimura, T.; Kubota, K.; Lai, E.C.; Man, K.K.; Nguyen, T.A.; Ooba, N.; et al. Variation in Association Between Thiazolidinediones and Heart Failure Across Ethnic Groups: Retrospective analysis of Large Healthcare Claims Databases in Six Countries. *Drug Saf.* **2015**, *38*, 823-831.
94. Roughead, E.E.; Kalisch, L.M.; Pratt, N.L.; Killer, G.; Barnard, A.; Gilbert, A.L. Managing glaucoma in those with co-morbidity: not as easy as it seems. *Ophthalmic Epidemiol.* **2012**, *19*, 74-82.
95. Roughead, E.E.; Pratt, N.L.; Kalisch Ellett, L.M.; Ramsay, E.N.; Barratt, J.D.; Morris, P.; Killer, G. Posttraumatic Stress Disorder, Antipsychotic Use and Risk of Dementia in Veterans. *J. Am. Geriatr. Soc.* **2017**, *65*, 1521-1526.
96. Roughead, E.E.; Ramsay, E.N.; Pratt, N.L.; Ryan, P.; Gilbert, A.L. Proton-pump inhibitors and the risk of antibiotic use and hospitalisation for pneumonia. *Med. J. Aust.* **2009**, *190*, 114-116.
97. Roxburgh, A.; Bruno, R.; Larance, B.; Burns, L. Prescription of opioid analgesics and related harms in Australia. *Med. J. Aust.* **2011**, *195*, 280-284.
98. Roxburgh, A.; Burns, L.; Drummer, O.H.; Pilgrim, J.; Farrell, M.; Degenhardt, L. Trends in fentanyl prescriptions and fentanyl-related mortality in Australia. *Drug Alcohol Rev* **2013**, *32*, 269–275.
99. Roxburgh, A.; Hall, W.D.; Dobbins, T.; Gisev, N.; Burns, L.; Pearson, S.; Degenhardt, L. Trends in heroin and pharmaceutical opioid overdose deaths in Australia. *Drug Alcohol Depend.* **2017**, *179*, 291-298.
100. Schaffer, A.L.; Zoega, H.; Tran, D.T.; Buckley, N.A.; Pearson, S.; Havard, A. Trajectories of antipsychotic use before and during pregnancy and associated maternal and birth characteristics. *Aust. N. Z. J. Psychiatry* **2019**, *53*, 1208-1221.

101. Stavrou, E.P.; Lu, C.Y.; Buckley, N.; Pearson, S. The role of comorbidities on the uptake of systemic treatment and 3-year survival in older cancer patients. *Ann. Oncol.* **2012**, *23*, 2422-2428.
102. Tang, M.; Schaffer, A.; Kiely, B.E.; Daniels, B.; Simes, R.J.; Lee, C.K.; Pearson, S.A. Treatment patterns and survival in HER2-positive early breast cancer: a whole-of-population Australian cohort study (2007-2016). *Br. J. Cancer* **2019**, *121*, 904-911.
103. Tran, D.T.; Preen, D.B.; Einarsdottir, K.; Kemp-Casey, A.; Randall, D.; Jorm, L.R.; Choi, S.K.Y.; Havard, A. Use of smoking cessation pharmacotherapies during pregnancy is not associated with increased risk of adverse pregnancy outcomes: a population-based cohort study. *BMC Med.* **2020**, *18*, 15.
104. Vitry, A.I.; Roughead, E.E.; Ramsay, E.N.; Preiss, A.K.; Ryan, P.; Gilbert, A.L.; Caughey, G.E.; Shakib, S.; Esterman, A.; Zhang, Y.; et al. Major bleeding risk associated with warfarin and co-medications in the elderly population. *Pharmacoepidemiol. Drug Saf.* **2011**, *20*, 1057-1063.
105. Wahab, I.A.; Pratt, N.L.; Ellett, L.K.; Roughead, E.E. Sequence Symmetry Analysis as a Signal Detection Tool for Potential Heart Failure Adverse Events in an Administrative Claims Database. *Drug Saf.* **2016**, *39*, 347-354.
106. Wahab, I.A.; Pratt, N.L.; Kalisch, L.M.; Roughead, E.E. Comparing time to adverse drug reaction signals in a spontaneous reporting database and a claims database: a case study of rofecoxib-induced myocardial infarction and rosiglitazone-induced heart failure signals in Australia. *Drug Saf.* **2014**, *37*, 53-64.
107. Zhan, C.; Roughead, E.; Liu, L.; Pratt, N.; Li, J. A data-driven method to detect adverse drug events from prescription data. *J. Biomed. Inform.* **2018**, *85*, 10-20.
